# A Novel Design of a Torsional Shape Memory Alloy Actuator for Active Rudder

**DOI:** 10.3390/s24154973

**Published:** 2024-07-31

**Authors:** Felipe S. Lima, Cícero R. Souto, Andersson G. Oliveira, Alysson D. Silvestre, Railson M. N. Alves, Sebastião E. S. Santos, Ricardo S. Gomez, Glauco R. F. Brito, André L. D. Bezerra, Diogenes S. M. Santana, Antonio G. B. Lima

**Affiliations:** 1 Department of Mechanical Engineering, Federal University of Paraíba, João Pessoa 58051-900, Brazil; felipe05silva06@gmail.com (F.S.L.); anderssonoliveira@gmail.com (A.G.O.); railson.nobrega@gmail.com (R.M.N.A.); 2 Department of Electrical Engineering, Federal University of Paraíba, João Pessoa 58051-900, Brazil; cicerosouto@cear.ufpb.br; 3 Department of Mechatronics, Federal Institute of Education, Science and Technology of Pernambuco, Caruaru 55040-120, Brazil; alyson.silvestre@cear.ufpb.br; 4 Department of Mechanical Engineering, Federal University of Campina Grande, Campina Grande 58429-900, Brazil; sebastianerailson@gmail.com (S.E.S.S.); antonio.gilson@ufcg.edu.br (A.G.B.L.); 5 Postgraduate Program in Process Engineering, Federal University of Campina Grande, Campina Grande 58429-900, Brazil; glaucoruben@hotmail.com (G.R.F.B.); dr.andreldb@gmail.com (A.L.D.B.); diogenessmedeiross@gmail.com (D.S.M.S.)

**Keywords:** shape memory alloys, torsional actuator, active rudder

## Abstract

SMA actuators are a group of lightweight actuators that offer advantages over conventional technology and allow for simple and compact solutions to the increasing demand for electrical actuation. In particular, an increasing number of SMA torsional actuator applications have been published recently due to their ability to supply rotational motion under load, resulting in advantages such as module simplification and the reduction of overall product weight. This paper presents the conceptual design, operating principle, experimental characterization and working performance of torsional actuators applicable in active rudder in aeronautics. The proposed application comprises a pair of SMA torsion springs, which bi-directionally actuate the actuator by Joule heating and natural cooling. The experimental results confirm the functionality of the torsion springs actuated device and show the rotation angle of the developed active rudder was about 30° at a heating current of 5 A. After the design and experiment, one of their chief drawbacks is their relatively slow operating speed in rudder positioning, but this can be improved by control strategy and small modifications to the actuator mechanism described in this work.

## 1. Introduction

Shape memory alloys (SMAs) have garnered significant interest from researchers across various disciplines due to their unique properties [1,2,3,4,5,6,7]. These functional smart materials, typically made of nickel and titanium (NiTi), exhibit two distinctive properties that are not found in most conventional metals or alloys: the shape memory effect (SME) and super/pseudoelasticity (SE). In SME, the alloy can recover its original shape by heating while in SE, the original shape can be recovered if the load is released. The peculiar properties of SMAs are related to reversible martensitic phase transformations, that is, solid-to-solid diffusionless processes between a crystallographically more-ordered phase, the austenite, and a crystallographically less-ordered phase, the martensite [8,9]. Typically, the austenite is stable at lower stresses and higher temperatures, while the martensite is stable at higher stresses and at lower temperatures.

During the shape recovery, they are able to develop large forces, a fact that makes them exceptional actuators. SMAs actuators are promising candidates as replacements for electrical motors or hydraulic systems in weight and space critical applications since they provide the highest energy density among all known active materials and systems [1,10,11,12]. Furthermore, the flexibility of SMAs allows us to build actuation components in different configurations and shapes, which allow them to be adapted to multiple applications. The SME-based various types of SMA actuators have been devised and widely utilized in diverse scientific and industrial fields such as aerospace [4,13,14], robotics [15], biomedical engineering [16], automotive engineering [3], and clothes [7,17].

In engineered mechanical systems, the requirement for rotational motion towards opposing moments is widespread. SMA components have been employed to create rotary actuators using two approaches: converting linear actuation to rotational motion and directly harnessing rotational motion [18,19]. The former approach utilizes the contractile properties of SMA wires, springs, or thin films, and can be manipulated to generate continuous or non-continuous rotation.

Among all the possible actuation solutions, the helical shape is one of the most frequently used, due to the number of parameters that can be adjusted, its ease of fabrication, and its compactness [18,20,21]. For instance, torsion springs effectively transfer power through the twisting of their coils, resulting in notable benefits including system simplification, efficient utilization of space, and overall reduction in product weight [22,23,24,25].

The initial concepts employing SMA torsion springs for rotary actuators were first reported in 1999. Yoshida et al. [26] presented a miniaturized robot composed of modular units capable of configuring into different 2D and 3D shapes. The system utilized a male and female connection mechanism, employing locking pins to secure the modules together. The design incorporated a pair of opposing SMA torsion springs, enabling a reduction in system size. However, drawbacks of this approach included limited torque and a restricted range of movement. In a 2013 study, Salerno et al. [27] created an anchoring frame specifically designed for intra-abdominal surgery. It was inserted through a natural orifice in natural orifice transluminal endoscopic surgery procedures. The shape memory alloy frame, initially inserted straight through the oral orifice using an endoscopic approach, transformed into a triangular shape when activated in vivo. This triangular configuration provided a larger area for magnetic coupling and a stable base for robot modules. The frame served as a platform for holding instrumentation or cameras, which could be wirelessly controlled by an external operator. In 2015, Sheng and Desai [28], followed by Sheng et al. [29], investigated a torsional actuator using torsional springs in antagonist configuration for a surgical robot prototype. This actuator was incorporated into a surgical robot prototype to showcase its performance in a humid environment while being guided by C-Arm CT imaging. The study revealed a rotation stroke of ±20° at a frequency of 0.05 Hz.

Recently, the development of new aeronautical structures and the implementation of innovative materials has been mandatory for succeeding in critical tasks in terms of weight, fuel consumption, aerodynamic efficiency, cost reduction, and so on [13,30,31,32]. Therefore, in many previous research studies, the SMA torsional actuators have been designed with various types of rotational driving mechanism as a significant part. For over 20 years, numerous studies have focused on the development of applications utilizing SMA torque tubes for morphing wings, both fixed and rotary [33,34,35,36,37,38,39,40,41]. In contrast to these, the development of a torsion actuator based on SMA torsion springs has not been an adequately investigated alternative for this field.

This work explores the merits of the design and an evaluation of an innovative torsional actuator driven by SMA torsion springs placed on a 3D-printed rudder structure. The proposed concept has two antagonistic SMA torsion springs, which can be actuated independently to obtain bidirectional movement. In what follows, for each SMA torsion spring, an approach to exploit stress-induced martensite (SIM) was used; this process is based on the phenomenon called high-performance shape memory effect (HP-SME) which allows the development of new actuators [42]. Furthermore, compared to other forms of SMA material, the SMA torsion spring allows a more compact design since it can directly generate a proper stroke. The remaining part of this article is organized as follows: Section 2 deals with the experimental setup developed to characterize the SMA torsion spring; this section also presents the mechanical design of the active rudder and the general internal interconnections. Section 3 discusses the experimental results and the potential application of this actuator, and, finally, some general concluding remarks are given in Section 4.

## 2. Methodology

### 2.1. Experimental Procedure

#### 2.1.1. Material and Spring Manufacturing

To perform the experiments, a NiTi SMA torsion spring was manufactured from a commercial NiTi wire with a diameter of 1.5 mm and a nominal composition of 56% Ni-Ti (%wt), supplied by SANDINOX Biomaterials (São Paulo, Brazil). The fabrication and the shape setting of the SMA helical springs are described by several authors [43,44,45,46,47]. The production of the springs used in this work is greatly inspired by these articles. Although any wire diameters would be used to design these torsional springs, due to the commercial availability of wire diameters, 1.5 mm were employed without affecting the general objective, once the prototype scale would be adjusted properly, regardless spring size.

**Figure 1:**
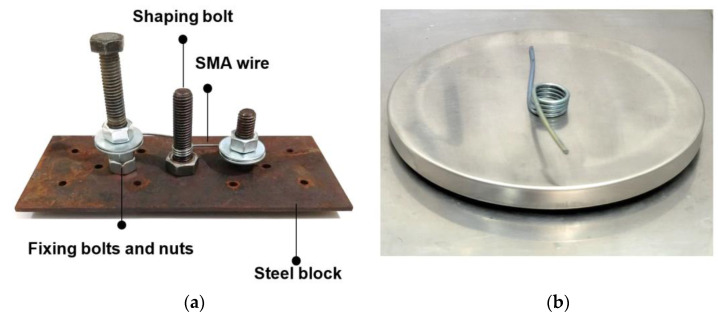
Fabrication method of the SMA springs: (**a**) SMA wire fixture used to keep the wire as a torsion spring during shape setting; (**b**) SMA torsion spring after shape setting.

There are two steps for training NiTi SMA torsion spring. First, The NiTi wires were fixed on the device shown in Figure 1a. It was further wound around an M10 bolt, and the spring was constrained into its desired shape. The two ends of the NiTi wire are fixed by bolts and nuts. The second step is the heat treatment of the torsion spring. A wide range of temperature from 300 °C to 900 °C may be chosen for the shape setting of NiTi. However, in order to optimize the combination of physical and mechanical properties, heat treating temperatures for binary NiTi alloys are usually chosen in the narrower range of 325 °C–525 °C. Heat treating times are typically 5–30 min [43,46].

The NiTi wire with the metallic block is kept at 500 °C in a furnace for 30 min, and then rapidly cooled in free air. After the annealing process, the SMA wire is removed from the metallic block and forms an SMA torsion spring which shows one-way shape memory. When the SMA torsion spring is deformed and then heated, it will tend to recover its original shape and exert a pulling force if its two ends are constrained. The finished spring may be seen in Figure 1b.

Based on well design practices, towards the manual fabrication process of the specimens, the basic characteristics of the SMA torsion spring are shown in Table 1 [23,24].

#### 2.1.2. Thermal Characterization of the NiTi Wire and NiTi Torsion Spring

In order to investigate the phase transformation behaviors of the SMA wires, a series of DSC (differential scanning calorimetry) tests were conducted. The tests were performed on a model Q20 from TA Instruments (New Castle, DE, USA). The temperature range was from −75 °C to 75 °C with a cooling/heating rate of 5 °C/min under a nitrogen atmosphere (ASTM F2004-17 [48]). Since the DSC technique is based on heat exchange, a small sample (weight of 40 mg) was required to ensure a uniform heat distribution during the tests. Therefore, the DSC analysis was performed on a sample taken from the wire material before the manufacturing process. The starting and final phase transformation temperatures, both during forward and reverse martensitic transformation, were obtained using the tangent intersection method applied to the DSC peaks.

**Figure 2:**
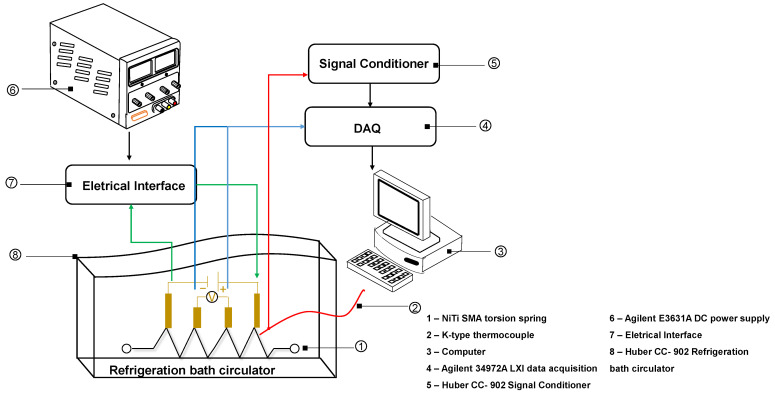
Schematic describing the conceptual ER experiment.

The phase transformation temperatures of the NiTi SMA torsion spring have been investigated by electrical resistance measurements (ER), a non-destructive technique that allows analysis of the entire torsion spring. ER measurements are carried out by a conventional four terminal DC method. During these measurements, a constant electrical current (i) is passed through the sample, and two conductive wires are spot-welded near the isolated ceramic grips of the extensometer to measure the voltage signal (∆V). The electrical current remains constant throughout the process. This concept tests in a SMA torsion spring, which is sketched in Figure 2.

The thermal cycling tests were carried out in the thermal controlled bath (model: CC-902, Huber, Germany). Besides, a K-type thermocouple was positioned at the center of the SMA torsion spring and linked in the signal conditioner and then to an acquisition system. The sample was conditioned in the refrigeration bath circulator. Conditioning took place by thermal cycling from −60 °C to 100 °C in steps of 5 °C/min, following experimental procedures as reported in the literature [49,50,51].

#### 2.1.3. Quasi-Static Characterization of the NiTi Torsion Spring

**Figure 3:**
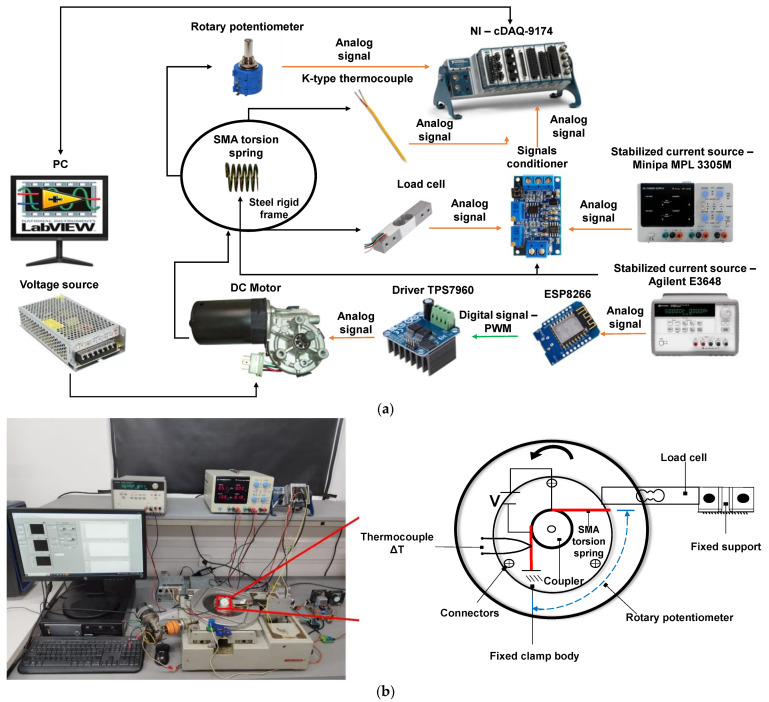
Experimental setup for the SMA torsion spring characterization: (**a**) Schematic outline of the experimental setup; (**b**) Photograph of the experimental setup and detail of the position sensor and SMA torsion spring (**right**).

Firstly, in order to understand the SMA-actuated torsion actuator’s mechanical performance. In this step, the characterization of the SMA torsion spring is obtained through the relationship between the pushing force and the torsion angle measured at room temperature. For this reason, an experimental setup is devised, as depicted in Figure 3a. The SMA torsion spring is connected to the load cell (CZL 635) with a maximum capacity of 50 N at its top coil. The bottom coil is fixed to the base plate on the platform. A DC motor is used to drive the base plate at the desired angle using the driver TPS7960. Accordingly, the SMA torsion spring loading can be performed by driving the motor at a constant rate, and blocked tests can be realized by halting the motor at various angles and activating the actuator. Additionally, the SMA torsion spring winds along the thread on the nonconductive couplings, the couplings were made of PTFE material which can tolerate the temperature up to 230 °C; as well, PTFE is not electrical conductor, ensuring total electrical isolation from the steel rigid frame.

To induce SMA phase transition, a pair of electrical wires are inserted to the top and bottom coils to generate Joule heating by the application of an electrical current of 5 A using a stabilized current source (Agilent E3648, Agilent Technologies, Santa Clara, CA, USA). Therefore, the temperature is monitored by the K type thermocouples. It is well known that the temperature distribution along the entire length of the SMA wire may not be homogeneous, partly depending on the connection terminals and the crimp.

The control of a direct current through Agilent E3648 is manually carried out, ensuring that the process is considered quasi-static and therefore that the temperature is gradually varied. The detailed study of heat distribution in the spring surface would be objective of toward studies. Yet, the objective of this instrumentation is to monitor the mean temperature during the actuator process. Thus, the thermocouples are soldered in the middle section of the torsion spring. The laboratory cooling took place through natural convection. Moreover, to obtain the temperature from the thermocouple, a monolithic thermocouple amplifier (in NI—CDAQ 9174) was used to make the output signal intelligible to the microcontroller and feed the temperature to the computer. The system consists of an adjustable angle sensor based on rotary potentiometer for capturing the SMA torsion spring movement (torsion angle). The voltage readings were taken directly from the potentiometer which was in series with the steel rigid frame and directly attached to it, providing a capture of all rotations. The measurement and control setup was based on components from national instruments and used LabVIEW^®^ 2022 software to control the experiments and data collection. Their signals were captured by the NI 9201 and NI 9213 data acquisition board. The measurement results were transmitted to the computer via a NI compact DAQ-9174. Considering the technical requirements, the experimental procedure operated with an angular speed of 3° s^−1^ to simulate quasi-static conditions and the data acquisition sampling frequency of 10 Hz.

Each experimental condition was made 10 actuations prior to each data collection. The atmosphere for all experiments was room temperature (20–22 °C). No structural damage was observed in the mechanical parts after tests.

#### 2.1.4. Design Concept of SMA Torsion Actuator

**Figure 4:**
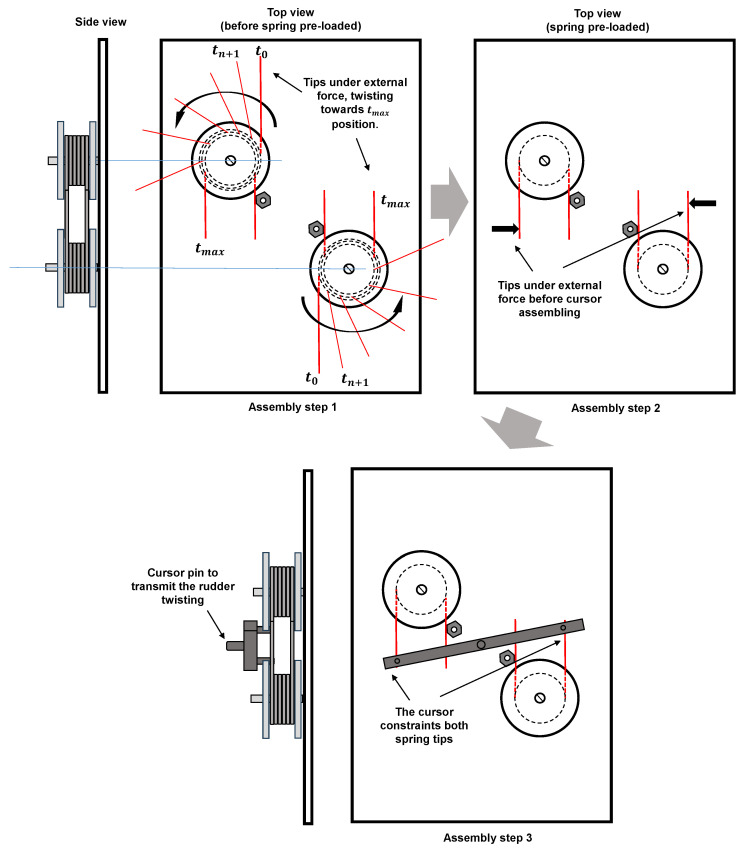
The general scheme of SMA springs assembling in the mechanism chassis and explanation of internal forces due to tip spring constrains, locked by mechanism.

This prototype considers a simplified model of an aeronautical rudder to represent the basic geometry of a rudder displacement mechanism, in this sense, employing a SMA actuator. The concept of a SMA torsion actuator is based on the opposed and combined movement of a SMA springs pair. Figure 4 depicts the general scheme of SMA springs assembling when attached in the mechanism chassis, and the explanation of internal forces due to tip spring constrains, locked by mechanism.

Figure 4 exhibits three assembly steps. In assembly step 1, the top view (helped by a side view for clarification about springs mounting) demonstrates the tip springs deformation around each spring coil from the initial and undeformed position (in time t_0_) to the final and deformed position (in time t_max_). In the final position, the restoring force due to elastic deformation takes place in each spring tip. During the assembling of the mechanism, no springs thermal are activated and it is kept in the environmental temperature. Because of this, the restoring elastic force is independent on the material phase transformation. In assembly step 2, is necessary to apply external forces under each spring tip to allow the third assembly step (the cursor fixturing). So, the restoring force of each spring may be interpreted as a preload stage for SMA springs. In assembly step three, the rigid cursor is attached in each spring tip, so that the mechanism equilibrates its internal forces and performs an offset neutral position due to this equilibrium. The neutral position takes place until the equilibrium between the internal forces from each spring exists. In the midspan of rigid cursor, the central pin is connected to the active rudder part. The degree of freedom allows the horizontal displacement of the rigid cursor so that the linear displacement of the cursor in torque to the active rudder is converted.

**Figure 5:**
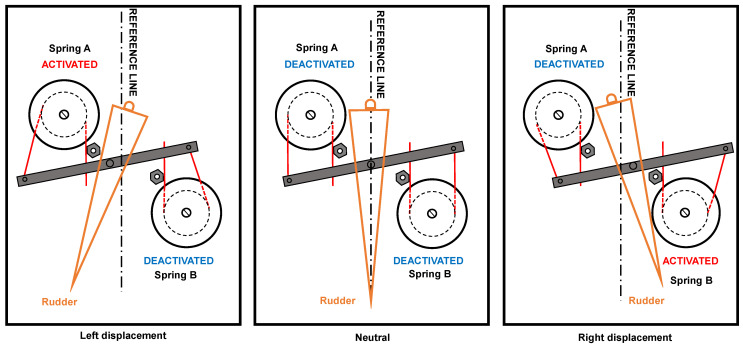
Top view of mechanism to demonstrate the converting lateral movement of cursor in rotational movement of active rudder.

The principle of mechanism operation when the SMA springs are activated is shown in Figure 5. In the neutral position, no springs are activated, and the internal forces keep the cursor in the central position. When spring A is activated, the material phase transformation initiates and the restoring force increases. The mechanism loses the equilibrium, and the resultant force pulls the cursor to the left position. When spring B is activated, the opposed moment takes place, and the cursor moves to the right. The electrical circuit employs relays to avoid the springs activation simultaneously. Yet, each time, only one spring performs movement while the opposed spring works as a bias spring in the mechanism. To general understanding of rudder movement, Figure 5 illustrates the conversion from the lateral movement of the cursor (due to springs operation) to the rotational moment of the rudder. The maximum rudder angle depends on SMA springs’ operation and the geometry of general mechanism.

#### 2.1.5. Fabrication of Functional Prototype and Experimental Setup

**Figure 6:**
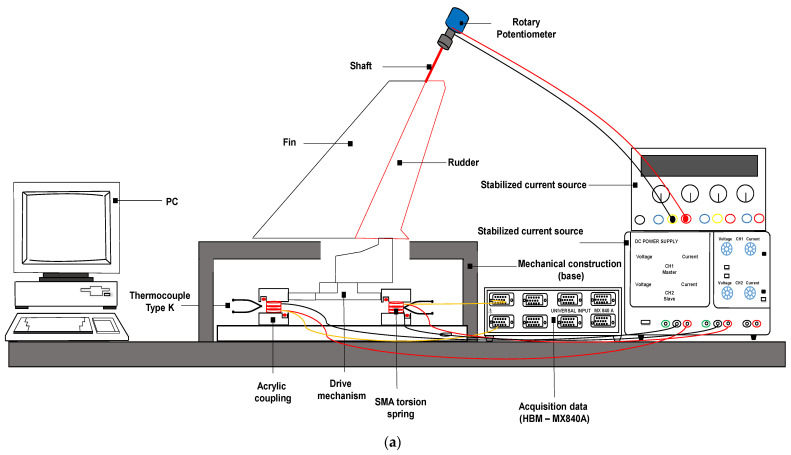

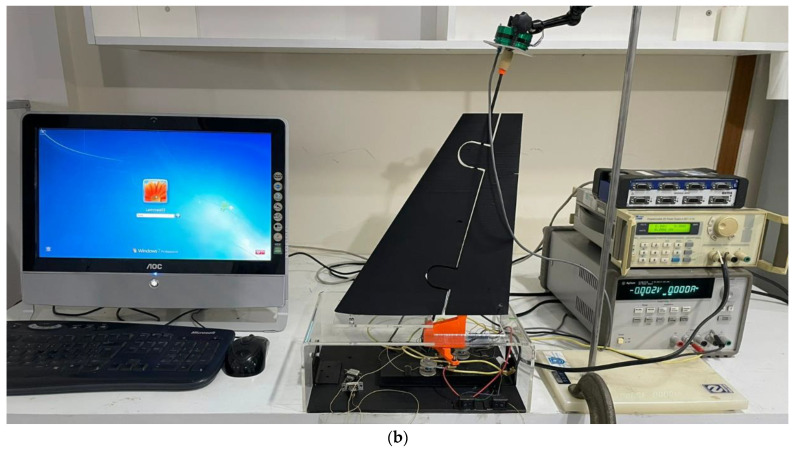
Functional test of the proposed smart rudder: (**a**) Schematic diagram of experimental setup; (**b**) Image of the experimental setup for the idealized performance tests.

The conceptually designed active rudder with SMA torsion springs is fabricated as a functional prototype in order to assess experimental evaluation of its working performance. The detail design of the active rudder was performed using a 3D CAD system (SolidWorks 2018) and it was fabricated by using a high-precision 3D printer. A NACA 0012 airfoil with a chord of 250 mm and span of 280 mm was designed. PETG plastic was selected as the material model as it can produce the desired deflection and mainly because both the properties and price are intermediate between the lower priced commodity thermoplastics and the more expensive high performance engineering plastic. Although an arbitrary airfoil shape is used in the following analyses, it is vital to note that these mechanisms can be adapted to any other airfoil shape, provided they can accommodate SMA torsion spring. Subsequently, metallic shafts and micro-bearings are implemented on the mechanism. Due to the absence of aerodynamic loads, the main opposing force against the rudder rotation is the internal friction of the mechanism. In practice, forward research may consider other external forces and adjust the spring dimensions for proper operation. To investigate the performance of the final 3D-printed SMA torsion spring-based active rudder, a set of experiments was conducted on a testing platform, as shown in Figure 6.

Specifically, the change in angular displacement pattern relative to the capacity of each SMA actuating unit was analyzed. As discussed in Section 2.1.4, only one SMA actuating unit should be activated to provide one particular direction, while the other actuating unit should be kept deactivated. For this purpose, when the applied current to the SMA actuating unit raises the temperature of the spring above the transformation threshold, the torsion spring contracts and produces the angular displacement of the rudder. A stabilized current source, Agilent E3633A, is used to supply Joule heating to the SMA torsion springs through a direct current (DC) pulse of 5 A, with manual shutdown (on-off control). The temperature is measured by the K-type thermocouples, which insert in the middle of the coil spring. To determine the maximum amplitude during the oscillation of the proposed design at each instant, the angular position of the rudder is sensed by the rotary potentiometer (100 Ω) attached at the rear end of the shaft. To demonstrate measurement validation and facilitate comparisons, a protractor is mounted to manually record the angular displacement. All signals were amplified, recorded, and analyzed using an 8-channel HBM Quantum MX840A measuring amplifier module and Catman Easy software version 3.5.1. The testing process was repeated for one hour, with each experimental condition replicated five times, and a sampling rate set at 150 Hz. The average of the five results was calculated and used as the final result.

## 3. Results

### 3.1. Experimental Analysis

#### 3.1.1. Transformation Temperatures

**Figure 7:**
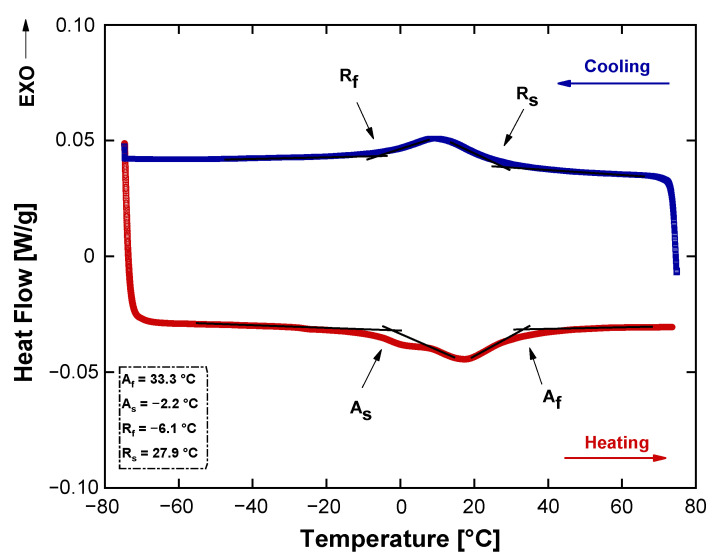
DSC results for a representative sample obtained from as-received NiTi wire.

The transformation temperatures for the as-received wire are shown in Figure 7. There are two peaks during the heating and cooling processes. At cooling, one exothermic transition is present corresponding to austenite—r-phase, defined respectively by a r-phase start transition temperature (Rs = 27.9 °C) and a r-phase finish transition temperature (Rf = −6.1 °C), indicating that the lifted off and thus stress free NiTi has a very narrow temperature hysteresis [52]. During the heating process, the DSC thermogram is characterized by a single-step due to the formation of an austenite phase defined by an austenite start temperature (As = −2.2 °C) and an austenite finish temperature (Af = 33.3 °C). The utilized DSC instrument was limited to the minimum temperature of −75 °C. Due to this temperature limitation, the determination of Ms and Mf for the sample was not possible. Based on these results, the presence of the r-phase indicates that the material had been annealed below the recrystallization temperature [53]. The DSC data also show that the as-received wire exhibit thermoelastic martensitic transformation. Hence, it is expected that the torsion spring will show the SME.

It is worth mentioning that, as shown by the DSC curve in the figure, a small secondary peak around 0 °C is observed on the heating curve, associated with the two-step transformation from martensite to austenite. The occurrence of this two-step transformation during heating may be related to a low annealing temperature. Huang et al. [54] observed the occurrence of two-stage reverse transformation (RT) in a Ti-50.85at.%Ni superelastic alloy annealed below 823 K, with the separation of the peaks being more evident at temperatures below 673 K. They noted that, at these temperatures, the RT peaks (from martensite to R-phase and from R-phase to austenite) do not overlap. Similarly, Lach et al. [55] and Shahmir et al. [56] also reported two-stage RT in low-temperature annealed Ni-Ti superelastic alloys.

**Figure 8:**
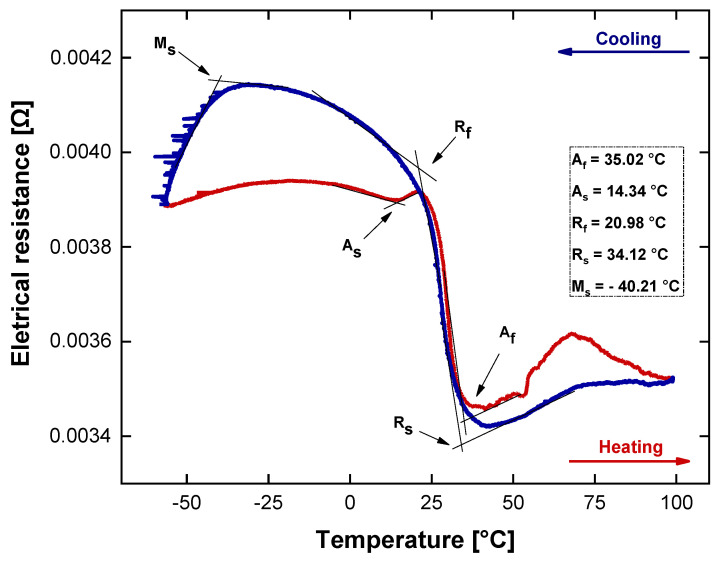
Measured electrical resistance of SMA torsion spring during heating and cooling cycles.

Based on the ER test, Figure 8 shows the change in the resistance of the spring as a function of temperature. During cooling, when resistivity increases, it is possible to evaluate r-phase start and finish temperatures. Moreover, the resistivity curve is characterized by the transition between r-phase—martensite, defined at lower temperatures by Ms. Upon the heating process, the martensite directly transforms to the austenite, without intermediate peaks. Compared to the calorimetric test, the phase transformation temperatures are slightly shifted to higher values due to thermomechanical procedure. From this curve, at a room temperature, the SMA torsion spring is partially martensitic and austenitic. Hence, only the r-phase transformation is used for actuation, which is of special interest.

#### 3.1.2. Quasi-Static Tests

A little-explored effect in literature is the shape memory effect involving tension-induced martensite. According to Casati et al. [42], this process is based on the phenomenon known as the high-performance shape memory effect (HP-SME), which enables the development of new shape memory actuators operating under high loads and temperatures conditions. Consequently, the SMA element can be driven actively and passively, changing only the load and temperature conditions. In this work, at room temperature, the springs remained in a mixed condition with a fraction of martensite and austenite present.

**Figure 9:**
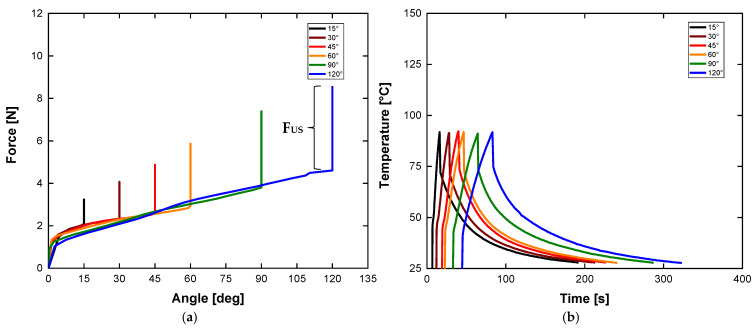
The experimental results for the force test: (**a**) Variation of the reaction force as a function of angle for an SMA torsion spring; (**b**) Temperature behavior measured on the actuator.

Considering this, force tests were performed, stretching torsion springs isothermally to 15°, 30°, 45°, 60°, 90°, and 120° angular displacements (Figure 9a). Subsequently, the actuator was heated under constant stress to a temperature (Figure 9b) to verify the maximum generated force. When the heating is removed, the spring returns to room temperature, reducing the force and returning to its initial state. The maximum useful force (Fus) was approximately 3.9 N and was generated in the temperature range between 27 °C and 95 °C (see Figure 9b). This force is responsible for the rotational movement of the active rudder. Observe that the available force Fus rises with the increasing of torsion angle, a characteristic directly related to the preload. Considering the paper presented by Sheng et al. [28], which relates the reaction force F [N] to the angle [°], the reported values were 0.1 N for a maximum angle of approximately 90° for a spring with a wire diameter of 0.5 mm and a spring diameter of 6.8 mm. In the current spring, with high geometric parameters, the reaction force for the same angle is around 4 N. The increase in coil diameter, along with the fraction of austenite present, helps to explain the increase in the resulting force values.

#### 3.1.3. Actuation Test of the Active Rudder

Conventional actuators, such as hydraulic and pneumatic are excessively heavy, causing cost loss. This problem can be solved by using the lightweight and reliable SMA actuators in primary structures that provide the same functionality.

For these experiments, the commands of the prototype are manual for activation and deactivation. Toward the evolution of this concept, control laws can be applied in order to optimize command and ensure more precision and controllability. For now, manual activation was enough to verify the mechanism’s feasibility.

**Figure 10:**
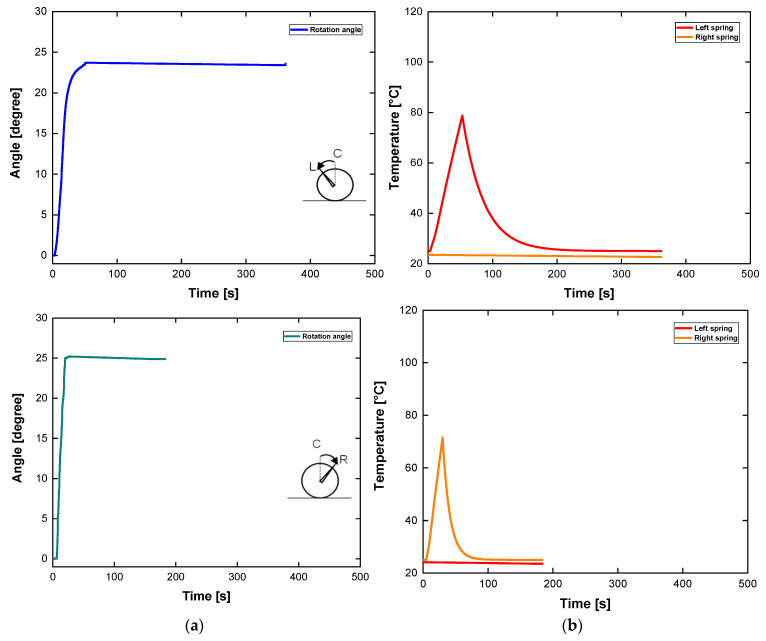
Active rudder experimental results (angle and temperature) with different unidirectional displacements: (**a**) curves of angle-time under step input of 5 A; (**b**) Corresponding thermal responses to the current pulses.

The time required for the displacement to reach the maximum value was recorded as the response time. The response times were 52 s and 32 s when an electric current intensity of 5 A was applied for the heating phases. From Figure 10a, it can be observed that the relationship between the maximum angular displacement and time based on experimental result is capable of providing a different performance. After reaching the maximum angle of 23.7° and 25.2° to the left and right, respectively, the input power was switched off manually. In addition, it takes a considerably long time to cool the heated SMA torsion springs to the ambient temperature using laminar natural convection of air.

**Figure 11:**
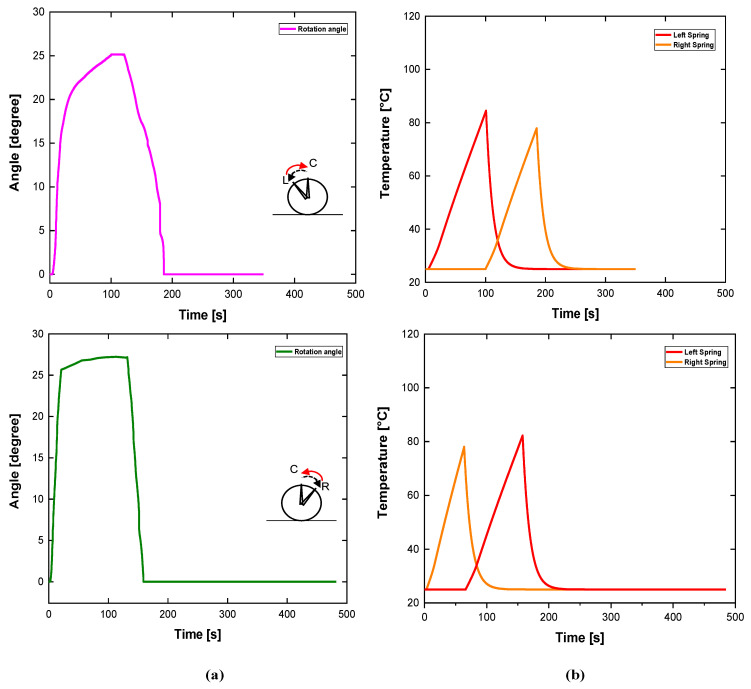
Active rudder experimental results (angle and temperature) with different bidirectional displacements: (**a**) curves of angle-time under step input of 5A; (**b**) Corresponding thermal responses to the current pulses.

In another test, the active rudder desired path was to follow a bidirectional motion. This test was performed to show the mechanism performance when following a smooth change in the desired direction. After optimization of the torsion spring installation process and some smaller design improvements, the resultant angular displacement to time relation during one period is shown in Figure 11a.

After reaching the maximum angular displacement of 25.5° in about 100 s, spring A (Figure 5) was deactivated while spring B was activated. This movement shifted the active rudder to the original position in 60 s. In the opposite direction, the maximum angular displacement was 27.5° in 100 s. In this direction, the return to the original position took place in 70 s. This asymmetry can be explained due to the different hysteresis of the torsional SMA springs. In addition, the initial deformation, necessary to attach the springs on the frame with mechanical friction, may change the overall mechanical behavior. It is important to emphasize that the return to zero angle is not natural after the end of the thermal cycle. Figure 11b depicts the thermal response to bidirectional movements. Based on these results it is observed that the restoring force due to the activated spring is only effective when this force overcomes the previous force generated by the opposite spring. This interaction depends on the simultaneous phase transformation of the activated spring and the cooling of the opposite element. After analyzing the behavior of the system, one potential approach to make this angular displacement more controllable is to adapt the feedback control.

The experimental results with one directional activation demonstrate the effectiveness of spring operation, its heating and cooling curves, the time response, and the maximum operational angle. Only the bidirectional experiment highlights a phenomenon related to interaction of two springs working simultaneously and may be similar to a real application. Figure 11a depicts the real rudder angular displacement as a result of an active spring. At the same time, Figure 11b depicts the curves of the heating and cooling spring of each actuator simultaneously. During the movement to the right (clockwise—from left to the center) the left spring starts its movement. During the initial seconds of its heating, the left spring observes a resistance force from the opposite spring. This resistance force is due to the residual heat presented in the opposite spring in the final step of its cooling.

The result of this mechanical resistance is a plateau in the top of angle curve. Considering the symmetry, when the opposite direction (counter-clockwise—from right to the center) a similar plateau is observed after left spring activation (in about 60 s). The overall time response depends on the thermomechanical parameters of device, since wire diameter, electrical current, thermal convection, and control system law (if employed), so the evolution concept would supply these concerns. The improvement of time response is an important parameter to ensure the viability of this concept in terms of use in rudder aeronautic systems and similar applications.

## 4. Conclusions

In this paper, an active rudder actuated by an SMA torsional actuator is proposed. For bidirectional motion capability, a driving mechanism designed with two torsion spring units is applied to the actuator. Based on the sequential activation of each spring, one spring unit is electrically heated to actuate the rudder in one direction, while the other unit is used to return to the initial position. The NiTi torsion spring with an Af of 35 °C (stress-free) generates a maximum useful force of 3.9 N, producing rotation angles of 30° to the right and 40° to the left on the rudder. More importantly, the restoring force of the right spring is only effective when its stiffness exceeds that of the left spring during cooling.

The compact size, kinematic simplicity, modularity of the design, and bidirectional capability make this actuator suitable for various scientific and industrial fields, exemplified by aeronautics. However, a critical issue with the presented active rudder is the long movement time, as the SMA torsion spring is naturally cooled in the air, resulting in low displacement efficiency. Additionally, the asymmetry in angle, response time, and temperature between each spring reinforces the need for future improvements to include a control law application in terms of heating, cooling, and positioning.

The low weight is a significant advantage in aeronautic applications. This prototype maintains the low-weight characteristic by employing a simple device with an actuator using a torsion spring. However, the electrical consumption might represent a drawback, which should be confirmed by comparing it with a hydraulic actuator for the same application. This suggests a comprehensive energy balance comparison between a hydraulic versus an electrical platform with SMA to analyze energy efficiency.

Our future research will focus on rapid cooling methods for the SMA torsion spring, associated with the metallurgical behavior of the alloy. Additionally, different types of control strategies will be explored to ensure higher precision in angular displacement.

## Figures and Tables

**Table 1:** SMA torsion springs parameters.

Parameter	Symbol	Value	Unit
Mass	m	2.02	g
Wire diameter	d	1.5	mm
Mean diameter	D	10.5	mm
No. of active coils	n	3	N/A
Spring index	-	7	N/A

## Data Availability

The data that support the findings of this study are available upon request from the authors.
